# Nursing home staff’s experiences and reflections regarding disordered eating among older adults: a focus group and vignette-based study

**DOI:** 10.1186/s40337-026-01668-w

**Published:** 2026-06-04

**Authors:** Mikaela Willmer, Maria Engström, David Conde Caballero, Lisa Ryan, Paula Conroy, Elisabeth Rothenberg, Annakarin Olsson

**Affiliations:** 1 Department of Caring Science, Faculty of Health and Occupational Studies, University of Gävle, Gävle, Sweden; 2 Interdisciplinary Group on Society, Culture and Health (GISCSA), Faculty of Nursing and Occupational Therapy, University of Extremadura, Cáceres, Spain; 3 School of Health, Sport Science and Nutrition, Atlantic Technological University, Galway, Ireland; 4 Department of Sport, Exercise and Nutrition, Atlantic Technological University, Galway, Ireland; 5 Department of Nursing and Health Sciences, Faculty of Health Science, Kristianstad University, Kristianstad, Sweden

**Keywords:** Eating disorders, Focus group, Healthcare staff, Older adults, Residential care

## Abstract

**Background:**

Eating disorders are often perceived to mainly affect young women, however previous research shows that they can both appear and persist in older populations. Research on eating disorders in older people is scarce, and there are no treatment guidelines or specialized diagnostic criteria, meaning that disordered eating in older adults is often misattributed to dementia, somatic illness, or the ageing process itself. This increases the risk of incorrect diagnosis and treatment, and hinders person-centred care. In order to start bridging this knowledge gap, the present study aimed to describe nursing home staff’s experiences and reflections on disordered eating among the residents.

**Methods:**

The study adopted a qualitative approach and a descriptive design. A total of 26 participants (registered nurses, licensed practical nurses and care assistants) were recruited from three nursing homes in mid-Sweden. Four focus group interviews were conducted, using a fictional patient case – a vignette – to help discussion. The interviews were audio recorded, transcribed, and analysed using qualitative content analysis inspired by Graneheim and Lundman.

**Results:**

Three themes were identified during the analysis. The theme *It’s not disordered eating behavior*,* it’s dementia or ageing – or is it?* referred to staff’s initial attribution of all problematic eating behaviors to dementia or other age-related illnesses. The theme *Lack of knowledge*,* lack of time*,* lack of resources – We don’t know*,* and no one else does*,* either* contained reflections on the lack of education and resources that was experienced in all professions. The theme *Navigating conflicts of interest related to problematic eating behaviors – a rocky path* included perceptions of conflicts and ethical dilemmas related to food and eating.

**Conclusions:**

Staff in nursing homes do not have sufficient training and resources to identify and diagnose residents with disordered eating behaviors, which is a barrier to safe and person-centred care.

## Abstract (plain language summary)

Not much is known about eating disorders (EDs) in older people, even though EDs affect all age groups. In this study, we carried out four focus group interviews with staff at three nursing homes in Sweden, and asked them to discuss and reflect on their experiences of disordered eating behaviors among the older people they work with. In the results, we found that the staff described disordered eating among the older people, but they often did not realize that the behaviors may be because of an ED. Instead, the staff thought that they might be caused by dementia, or by other illnesses related to the ageing process. The results also showed that nursing home staff lacked education and resources to deal with EDs among the residents, and that they didn’t know where to turn for help. They also described conflicts and ethical dilemmas around food and eating. It is important to improve nursing staff’s knowledge about EDs in older people, and to make sure there is access to suitable resources and specialized care.

## Background

Aside from the clinical eating disorders (EDs), which are listed in *Diagnostic and Statistical Manual of Mental Disorders* (DSM-5) [1] and have their own criteria, it has also been established that a number of individuals, perhaps as many as 10% of the adult female population, live with subclinical eating disorders, displaying some but not all characteristics of a clinical ED [2, 3]. Further down on the spectrum of severity, an even larger number of people experience some disordered eating behaviors. Whilst there is no consensus on the definition of disordered eating behaviors, the American National Association of Eating Disorders describes them as: “*a spectrum of problematic eating behaviors and distorted attitudes towards food*,* weight*,* shape*,* and appearance”* [4]. These may include long-term restriction of food intake (also known as “chronic dieting” [5]), obsessive calorie-counting, or strong preoccupation with thoughts about weight, looks, or food intake. Clinical EDs, subclinical EDs and disordered eating behaviors all share the same core psychological and cognitive symptoms, namely a strong preoccupation with eating, weight and shape, strong body image concerns, and over-evaluation of the importance of body shape, weight and appearance [1].

Eating disorders are often perceived as primarily affecting adolescents and young adults, especially white women [6, 7]. However, older people can also be affected. Most commonly, older people with EDs have experienced onset at a younger age [8], but a study by Mangweth-Matzek et al. [9] found that EDs may also emerge in older adults, with a number of participants reporting onset after the age of 65.

It is difficult to estimate the prevalence of specific EDs in ageing populations, due to the scarcity of research and the significant variation in age range, gender, methodology and sample size in the studies that do exist [10]. Anorexia Nervosa seems to be the least common ED diagnosis in older populations, followed by Bulimia Nervosa and Binge Eating Disorder, which is the most common form of ED both in the general population and in older populations [8, 11].

A 2019 review found only five relevant epidemiological studies, and three of these also included individuals under the age of 65 [12]. The findings showed that clinical EDs as well as subclinical EDs and disordered eating behaviors are indeed present in the older population, with numbers ranging from 1.5% up to 6% in one included study [12]. Similarly, a 2021 systematic review attempting to summarize all previous research on the treatment of EDs in older people found only case studies and case series [13], meaning that there are currently no guidelines or best practices in existence for this age group.

The lack of research on EDs in older people is worrying, since identifying and supporting older individuals with clinical/subclinical EDs or disordered eating behaviors require a nuanced understanding of the unique physiological, psychological, and social challenges faced by this demographic [12]. This underscores the need for increased awareness and tailored diagnostic criteria for this age group. In order to effectively support older adults with clinical/subclinical EDs or disordered eating behaviors, healthcare professionals need to provide person-centred care (PCC). This emphasizes tailoring support to the individual’s unique needs, preferences, and circumstances, fostering a sense of autonomy, partnership and dignity [14].

The present study addresses this gap in the Swedish context. Sweden is divided into 290 municipalities, which are legally required to offer and maintain residential care facilities (also known as nursing homes) for older individuals who are no longer able to live independently and safely in their homes. Common reasons for needing to move into a residential care facility include different kinds of dementia, other cognitive impairments, or somatic illness. In 2021, 84,000 people over the age of 65 were living in a residential care facility [15].

Typically, the residents live in their own small apartments, with access to round-the-clock care. Meals are often cooked in large-scale industrial kitchens off-site, and then delivered to the residential facility where they are heated and served by the care staff. There are common areas for eating, socializing and taking part in organized activities. The care work is mostly carried out by assistant nurses or licensed practical nurses, with registered nurses (RNs) taking responsibility for planning, organizing, and directing the care work [16]. The Swedish National Board of Health and Welfare carries out regular surveys of residential care facilities, and these show that the proportion of care staff with adequate education is steadily decreasing, and was down to 76% in 2023. In the same year, there were on average 4.7 RNs per 100 residents on weekdays, and 0.6 RNs per 100 residents on weekends [17]. Person-centred care has been linked to improved quality of life among residents in residential care homes [18] In the PCC framework, staff competence as well as organizational support are clearly emphasized [19].

Looking from Sweden to the EU, publicly funded formal care is usually available across OECD countries but differs widely in terms of its scope and reach. An average of 11.5% of people aged 65 and older received long-term care in 2021 [20]. Similarly, across OECD countries 1.8% of the gross domestic product was allocated to long-term care on average, ranging from more than 3% of GDP in the Netherlands and the Nordic countries to around 0.5% of GDP or less in Greece, Poland and Latvia. This variation partly mirrors differences in the population structure but mostly reflects the stage of development of formal care systems, as opposed to more informal arrangements based mainly on care provided by unpaid family members [20].

Outside Europe, it may be noted that Australia has a similar system to the Nordic countries, with subsidized residential care homes for older people who can no longer live independently at home. The system is based on approved permanent residential care for those eligible according to a care needs assessment. It includes accommodation, personal care and access to nursing 24 h a day, as well as general health care, social activities and everyday living services (e.g., catering). Government-funded residential aged care is delivered by providers who are registered with the Aged Care Quality and Safety Commission (ACQSC) at Category 6. Residential care homes must also be approved by the ACQSC [21].

Biological changes due to ageing, such as reduced metabolic rate, diminished muscle mass, and altered taste and smell, can significantly impact nutritional intake and appetite [22]. The concept of Anorexia of Ageing, first described by Morley and Silver as early as in 1988, refers to the phenomenon of older people experiencing loss of appetite and reduced food intake in later life [23]. Additionally, chronic illnesses, medication side effects, and dental problems may cause changes in taste and smell, lack of appetite, and involuntary weight loss [24]. Lack of motivation to eat, atypical appetitive cues and sensory-driven factors in eating avoidance are prominent in the eating disorder ARFID [25]; diagnostic overshadowing of ARFID or other eating disorders or disordered eating may therefore occur due to ageing processes and presence of chronic health conditions [26]. Psychologically, older adults may experience significant life transitions, including retirement, loss of a spouse, or social isolation, which can contribute to the development of anxiety, depression, and subsequent eating disorders [27]. The interplay of these psychological stressors with pre-existing or newly developed mental health issues can worsen tendencies toward EDs [8, 28]. The cultural and generational differences in perceptions of body image and weight may also impact older adults differently compared to younger populations, necessitating a tailored approach to understanding and addressing their specific needs [29].

The clinical features of EDs in older people have been found to be similar to those in younger patients [30]. As stated above, EDs in older people may have persisted from their onset earlier in life, which appears to be the most common scenario, or they may have emerged later in life [8], with menopause seemingly a risk period for both exacerbations of existing EDs and emergence of new EDs [8]. When it comes to understanding the roles and possible interplay of commonly age-related features of EDs, such as changes in appetite and medication side effects, in relation to core aspects of EDs, such as body image concerns and fear of weight gain, research on older populations is still too sparse to be able to offer any real insights.

The stigma surrounding mental ill-health in older adults often leads to underreporting and underdiagnosis of psychiatric disorders, thereby impeding timely and effective intervention [30].

Research on healthcare staff’s experiences and knowledge of disordered eating behaviors among older people in residential care seems to be non-existent, despite its importance to ensure PCC for this vulnerable group of older adults. Thus, the present study is an attempt to increase knowledge about this research area, by describing the experiences and reflections of healthcare professionals regarding disordered eating behaviors among older adults in residential care settings in Sweden.

## Methods

### Aim

The aim of the study was to describe the experiences and reflections of healthcare professionals regarding disordered eating behaviors among older adults in residential care settings.

### Design

The present study adopted a qualitative approach and a descriptive design.

### Setting and participants

Recruitment of participants was conducted through convenience sampling [31]. Initial contact was made via the managers of three residential care facilities in mid-Sweden, consisting of 122, 62, and 48 single-occupancy apartments, respectively. Members of staff were recruited using the following inclusion criteria: minimum of six months’ experience of giving hands-on care to residents in residential care facilities to ensure familiarity with the working conditions and the care environment. The study targeted all healthcare professionals working in residential care facilities for older adults, including licensed practical nurses, assistant nurses, and registered nurses. Care co-ordinators and first-line managers were also welcomed to take part, as long as they had previously worked with delivering care in close contact with residents.

A total of 26 participants were recruited. Out of these, 20 were licensed practical nurses or assistant nurses, 4 were registered nurses, and 2 were currently working in managerial positions, although they both had long previous experience of hands-on care work.

### Data collection and procedure

Data collection involved conducting four focus group interviews at the three participating nursing homes (with two different groups in one of the nursing homes), from November 2023 to January 2024. The interviews were held at the participants’ workplaces to facilitate convenience and comfort. The focus groups included 4, 5, 6, and 11 participants, respectively [32]. Each focus group interview was audio recorded and lasted between 31 and 46 min, with an average of 38 min. Two researchers were present during the data collection. One researcher acted as the moderator (MW), guiding the discussion, and ensuring that all participants had the opportunity to contribute. The second researcher (AO) acted as a co-moderator and took notes on non-verbal cues and group dynamics, supplementing the audio recordings [33].

Following approval from the managers of the nursing homes, a suitable date and time for the interviews was arranged. The managers provided the staff with preliminary information about the study, and those who were interested in participation turned up. Before the start of the focus group interview, participants received oral and written information about the study, and signed a consent form. At the start of the session, the participants were provided with a brief, fictitious case study (vignette) describing an older person in residential care exhibiting behaviors suggestive of an eating disorder [34]. The vignette was developed by the first author based on her experience as a researcher and a dietitian. The vignette was used in a postgraduate course for nurses working in municipal care (mainly with older adults in residential care), and the nurses taking the course were asked to give input on the vignette and talk about whether it was relevant to their experiences. It was adjusted according to their comments, and further discussed with the co-authors, including ER, a registered dietitian with extensive clinical and research experience of older adults in residential care.

This vignette served as a starting point for the discussion, prompting participants to share their perceptions and experiences related to disordered eating behaviors among the older adults in their care. In addition to this, an interview guide was constructed and questions related to the vignette were asked, such as *Can you describe any similar cases from your own experiences of caring for older people in residential care?* and *What kind of support and education do you feel that you require in order to better be able to identify and care for residents with eating disorders?* Additional follow-up questions were asked to further deepen the researchers’ understanding, e.g., *Could you give some examples? Are you able to elaborate on that reflection?*

As described in the Introduction, problematic eating behaviors occur on a spectrum, from clinical EDs to less severe disordered eating behaviors that nonetheless cause the affected individual significant distress. The participants were not given any information about what constitutes an ED, nor were they asked to rank or differentiate between different types of clinical/subclinical EDs and disordered eating behaviors. Whatever the participants themselves felt constituted examples of EDs or disordered eating behaviors were acknowledged and discussed. Thus, some participants talked about “eating disorders” and then went on to describe behavior that would, to someone well-versed in the area, be defined as disordered eating behavior, or the other way around.

### Data analysis

The audio recordings were transcribed verbatim and analysed using qualitative content analysis inspired by Graneheim and Lundman [35]. Initially, meaning units, which included relevant words and sentences from the interviews, were identified based on the study aim. These meaning units were subsequently condensed to reduce the volume of text. The condensed meaning units were then assigned a code that succinctly described the text. The codes were then examined for differences and similarities, leading to the creation of subcategories. This process constituted the manifest part of the analysis [35, 36]. In the interpretation of subcategories, themes were identified. Reflections and discussions were conducted to reach a consensus on the themes [37]. Although described linearly, the analysis was an iterative process involving continuous movement between the whole and its parts [35]. Field notes from the interviews were also utilized in the analysis to enhance our understanding of the data. Confidentiality was ensured by using fictitious names for individuals and locations in the transcriptions. The co-moderator’s observations enriched the data analysis process, providing a more comprehensive understanding of the participants’ behaviors and interactions. This offered insights into the participants’ body language, facial expressions, and group dynamics, which were critical for interpreting verbal responses accurately. The observations also helped identifying participants’ underlying sentiments or tensions during the interviews. Furthermore, non-verbal cues and group interactions guided the identification of themes during analysis, ensuring that the analysis captured the full spectrum of the participants’ experiences and reflections [38].

It is important to be aware of and reflect on researcher preconceptions when conducting and analysing focus group interviews. In the present study, the first author (MW) is a registered dietitian with previous research and clinical experience of individuals with clinical/subclinical eating disorders and disordered eating behaviors. The last author (AKO) is a registered nurse with previous research and clinical experience of working with older adults in various care settings.

The analysis finally resulted in three themes, capturing the experiences and reflections of nursing home staff regarding disordered eating among the residents.

### Ethical considerations

Ethical approval was sought from the Swedish Ethical Review Authority (2023-03964-01), and an advisory opinion was issued stating that the present study did not require ethical permission under Swedish law. The participants signed an informed consent before the data collection started, ensuring they understood the purpose, procedures, and potential risks of the study. Confidentiality was strictly maintained, with all personal identifiers removed from the transcripts and findings.

Despite the Ethical Review Authority’s decision that an ethical approval was not required for the present study, there was still a possibility that the participants may experience negative effects of taking part. For example, the insight that the older people in their care may suffer from untreated eating disorders may cause the staff distress and doubts regarding their own professional abilities. The researchers responsible for the data collection took care to outline the nature of the study and the questions that would be asked, and emphasized that participants were free to talk as much or as little as they wished, and to leave the interview at any time.

The study reporting was guided by the Consolidated Criteria for Reporting Qualitative Studies (COREQ) checklist [39].

## Results

The analysis resulted in three themes: [1] *“It’s not disordered eating*,* it’s dementia or ageing – or is it?”* [2], *“Lack of knowledge*,* lack of time*,* lack of resources – We don’t know*,* and no one else does*,* either*”, and [3] “*Navigating conflicts of interest related to problematic eating behaviors – a rocky path*”.

Quotes from the different focus group interviews (FG 1 to 4) are presented to exemplify the different themes.

It’s not disordered eating behavior, it’s dementia or ageing – or is it?

This theme contains reflections related to the misattribution of possible ED-related behaviors and symptoms to other conditions or illnesses. In the early stages of the interviews, there was a general perception amongst the participants that any irregular or disordered eating behaviors displayed by the residents were either because of dementia, or because of the ageing process itself. The idea that some residents may have an eating disorder was either questioned, or outright rejected, with one of the participants saying:

*“We don’t have a lot of eating disorders here*,* that I know of. It’s more that when they don’t have an appetite*,* they’re pretty sick*,* pretty poorly*,* or pretty old.” (FG 1)*.

The participants ascribed both lack of appetite, excessive appetite, weight loss, weight gain, and dislike for the foods on offer to dementia or ageing. Several participants voiced the belief that dementia caused residents to binge eat: *“Participant A: We have another one who doesn’t have a sense of satiety now. She thinks that there isn’t enough food*,* so we have to put away the serving dishes. [makes a panting noise] sounds like that when she walks. Participant B: Yes*,* but that’s common with dementia*,* that you have … the sense of satiety disappears.” (FG 1)*.

As the participants discussed and shared their reflections on the eating behaviors of the residents, they realized that many of them had encountered residents who had a long history of dieting and who regularly voiced concerns about weight gain and becoming too heavy. They also felt that this life-long behavior of dietary restriction was so deeply rooted in the older individual’s personality that it was unaltered even by severe dementia.

*“It’s hard with those clients who come here and they have a behavior of having dieted their whole lives and … it’s so manifest in those people. … Those people*,* I think it’s hard to get them to eat*,* in the early stages of dementia but also at the end*,* because it’s a behavior that is so firmly rooted in them.” (FG 2)*.

Through their reflections, the participants gradually came to realize that the older people might *both* have dementia, *and* still suffer from life-long, ingrained beliefs and body image ideals.

*“Participant A: We have a lady with dementia here who comments a lot … and it’s like*,* she’s very particular*,* she likes to look good and she puts on face cream every day*,* paints her eyebrows and things*,* she’s very particular. But she doesn’t think about her weight*,* I don’t think. But it’s important to be thin*,* ‘I’ve always been petite’*,* she says. … Participant B: Oh yes*,* you often hear that comment.” (FG 2)*.

There was a growing insight amongst the participants that some of the older people in their care may display signs of eating disorders, and that these may have been misinterpreted as symptoms of dementia. In some cases, this was almost regarded as a revelation and as something that would change the participants’ future care. Several participants stated that they would think differently about the residents’ eating behavior in the future:

*“Participant A: …she’s been out running a lot in the evenings*,* before … I mean*,* in the past. She has been horseback riding*,* she’s done a lot of sports and she has been very active. And then I think there’s a divorce behind it all. Participant B: Maybe depression or something. … Participant A: Yes*,* in her life. So she could have been a bit like that before*,* like ‘oh no*,* I have to go out again now because I’ve eaten too much’ or something*,* or … Participant B: Yes*,* exactly. That you restrict so that you won’t gain weight and to conform to an ideal. I suppose that’s it. That’s why … you separate it*,* if you don’t eat because of illness or dementia or something like that. Yes. And an eating disorder. Participant A: We’re really going to pay more attention to it.” (FG 1)*.

*“But I’m going to think differently about her now [resident with binge eating behavior]. Yes*,* actually*,* I’ve never reflected on that before.” (FG 2).*

Lack of knowledge, lack of time, lack of resources – We don’t know, and no one else does, either.

This theme contains reflections on the perceived lack of knowledge and training on disordered eating behaviors, which was felt to permeate the entire care context, on all levels and for all professions. The participants voiced concerns that they did not know anything about eating disorders. They felt that they would be unable to tell the difference between signs of an eating disorder and signs of other physical or mental illnesses, and that the residents rarely arrived with complete information about their health history. The participants who were care assistants or licensed practical nurses stated that they would turn to the registered nurses for advice and support if they encountered a resident with disordered eating behaviors. However, the registered nurses admitted that they themselves also felt uncertain and ill-prepared to give the required support. When asked where she would turn for support, one of the registered nurses said:

*“Yes*,* but I suppose that would be the dietitian that you could ask*,* maybe … And then you’d have to look for it*,* what to … where to turn. And discuss*,* perhaps*,* in a group with the person in question and hear if you could … I don’t know.” (FG 4)*.

Another registered nurse said:

*“Most residents are frail and have many illnesses and underlying conditions that contribute to reduced appetite and that’s what … there’s a huge grey area. And being able to say which is which*,* is what I’m thinking.” (FG 4).*

She also mentioned that her nursing education had not contained very much information on eating disorders, or on mental ill-health in general.

The participants rarely brought up contact with physicians, but in one interview, it was mentioned that they didn’t seem well-educated on eating disorders, either

*“Participant A: I think this is something that will be more common in the future. Participant B: But I think it’s here now*,* too*,* but we don’t notice it. Participant A: Yes*,* that’s possible too*,* that we don’t see the signs and we don’t quite understand what it is. Participant C: It doesn’t seem like our physicians quite know it either [laughter]” (FG 1).*

One of the participants, a licensed practical nurse, shared a reflection that she had, in fact, suspected that some of the residents under her care were suffering from eating disorders, and that she had identified both restriction and binge eating behaviors. She also shared that she had previously had a friend who suffered from an eating disorder, and that this had given her insight and knowledge into the signs and symptoms.

It was a common reflection amongst the participants that once an older person enters residential care, they are lumped together into one, homogenous group of “geriatric patients”, meaning that their age and residential status were allowed to do an “override” on any previous, individual care needs the older person might have had before entering residential care.

This was especially felt to be the case when it came to mental ill-health, and they also stated that they had no access to a dietitian.

*“So*,* the problem is that we don’t have access to a dietitian. So if we notice symptoms*,* we try to investigate … put interventions in place. And then if that doesn’t help we can consult with a physician*,* a primary care physician. And a lot of the time we’re told that yes*,* but that’s age-related.” (FG 1)*.

*“It’s a bit unfair when you move into a nursing home*,* because you lose all other access*,* I think*,* to seeing a counsellor*,* you have … here*,* you are stopped by our nurses or by the primary care physician most of the time. You don’t have the same access to support for mental ill-health*,* because here you are an elderly person and all elderly people are in the same group.” (FG 2)*.

When the participants reflected on what kind of support they wanted, they mentioned advice and help from dietitians and mental health professionals as the most useful. *“Participant A: Yes*,* but absolutely access to a dietitian*,* it would be a great relief to be able to discuss with. Participant B: And to have some kind of action plan or something from a dietitian*,* some tips and advice.” (FG 1)*.

They also felt that more time would be required in order to get to the bottom of the reasons for the older person’s eating behavior, especially in those cases where the older person also had a dementia diagnosis.

*“Yes*,* it takes a lot of time*,* too*,* and a conversation … you might have ten minutes for this conversation and maybe you need an hour and a half. You might have to do some observations and … it’s time-consuming and we don’t really have those resources.” (FG 2)*.

### Navigating conflicts of interest related to problematic eating behaviors – a rocky path

In the third and final theme, there are reflections on the many dilemmas and conflicts that the participants perceived in relation to food and eating. The participants described their experiences related to different types of dilemmas or conflicts when it came to food and eating. These conflicts could play out between the older person themselves and the staff, thereby obscuring potential eating pathology and acting as a barrier to healthcare professionals’ recognition of clinically significant eating disorders in this population. In many cases, these conflicts created difficult ethical dilemmas and considerations for the staff. For example, they described a person with dementia who had lost her sense of satiety and wanted to keep eating large amounts of food. One person described it as

*“And in the end we were told not to give … we had to limit her meals. But she was constantly hungry. She wasn’t satisfied. And that doesn’t feel right*,* either.” (FG 2)*.

There were also recurrent descriptions of dilemmas when residents did not want to eat, and the staff described several different strategies to tempt, convince or even trick the older people to eat. These included adding sugar to the food to enhance the flavour, adding butter and cream, repeatedly offering food to residents who refused to eat, trying many different kinds of food, and giving the residents a special appetite-enhancing drink before meals. Again, some of the staff reflected on ethical concerns regarding some of these interventions, as they felt that they constituted an impeachment of the residents’ autonomy. This was described by one of the participants:

*“And then to give medicines*,* to get more of an appetite and that*,* and they have an eating disorder*,* are you allowed to do that? I mean*,* the person has to agree to it*,* if they’re not completely … if they have some kind of dementia so they can’t decide for themselves.” (FG 1)*.

Participants in one of the focus groups described how the husband of a resident repeatedly told her to stop eating, because he thought that she had gained too much weight and was getting fat. They also described a visit from one of the residents’ children:

*“Participant A: Yes. And this thing with relatives*,* ‘Oh*,* you have really gained weight now*,* Dad’*,* or Mum. It’s really hard to handle as a member of staff. You can’t tell someone: ‘No*,* you can’t say that’. Participant B: ’Stop saying that.’ No*,* you can’t. Participant A: Because we’ve experienced that*,* when you see that the resident is upset by it*,* but … and hurt.” (FG 2)*.

One of the licensed practical nurses also described how the husband of one resident came to visit and kept telling the resident that she was too big and should only have one biscuit with her tea. Even though the woman was diagnosed with dementia, the husband’s words clearly made her upset, and resulted in her touching and feeling her stomach for the rest of that day, voicing concerns that she was too big.

*“Yes*,* yes*,* when the relatives have visited*,* it’s really a lot of touching the belly.” (FG 2)*.

Sometimes, the residents themselves also worried about needing larger clothes, and the staff then tried to calm them by saying that they just needed larger clothes in order to be comfortable in their wheelchair, or for the incontinence pad to fit in their trousers.

The participants also described conflicts between those members of staff who were present during mealtimes and knew the residents well (mainly licensed practical nurses) and those who were in decision-making positions further removed from day-to-day care (mainly registered nurses and physicians). Whilst the registered nurses prescribed extra cream, butter and energy-dense food, the licensed practical nurses were sometimes unwilling to implement this, if they felt that the older person in question had a problematic relationship with food or would be unwilling to eat the extra calories.

“*Participant A: But they notice with their clothes when they gain weight*,* that the clothes get tight. So they will tell you that. Participant B: Yes*,* because the nurse tells you: ’it’s time to fortify with some cream and nutritional drinks’ because the resident has lost weight*,* because they [the nurses] aren’t there on the wards where they might hear all of this. The talk around the table at mealtimes*,* ’How much did you weigh today? Oh dear*,* oh dear.’ Participant C: Yes … it can turn into a competition who eats the least … ‘Oh*,* you’re taking a whole potato*,* then I’m going to take three quarters of a potato*,* and you will take half a potato’.” (FG 2)*.

## Discussion

Three themes were identified in the results of the present vignette-based focus group study. The theme “*It’s not disordered eating behavior*,* it’s dementia or ageing – or is it?”* showed that even though participants were initially prone to express that all problematic eating behaviors among the residents could be attributed to dementia or to the ageing process itself, they quickly realized during their discussion that some residents may display disordered eating. The theme *“Lack of knowledge*,* lack of time*,* lack of resources – We don’t know*,* and no one else does*,* either”* illustrated the perceived limitations in resources such as knowledge and education about eating disorders, which was felt to reach across all professions and levels of the organization. Finally, the theme *“Navigating conflicts of interest related to problematic eating behaviors – a rocky path”* contained reflections on the many conflicts that staff, residents and relatives became involved in related to food and eating.

The findings suggest that disordered eating behaviors are present in residential care facilities, yet staff reported insufficient training to identify these behaviors or initiate appropriate care. Management of the issue was frequently deferred within the professional hierarchy, from licensed practical nurses to registered nurses and further to physicians. Simultaneously, staff acknowledged limited resources and expressed low confidence in physicians’ ability to address the problem. These challenges appear to be primarily related to a lack of knowledge and education regarding disordered eating. Staff also expressed frustration with the limited access to dietitians and mental health personnel to consult, something the staff felt was unfair and detrimental to the residents’ health and well-being, and not in line with Swedish regulations. Swedish healthcare legislation stipulates that care should be given according to each patient’s needs, regardless of age, gender, ethnicity, sexual orientation, etc [40]. It could be argued that nursing home residents do not always receive equal opportunities and access to care compared to those living in their own home.

Clinical and subclinical disorders and disordered eating behaviors among the elderly need to be recognized, and guidelines for identification and treatment need to be developed and integrated with PCC. According to the framework on PCC, at the macro level, policy frameworks and strategic leadership are required that raise the issue [14]. At the prerequisite level, clarity in values, beliefs and skills are required to identify and manage EDs/disordered eating behaviors. In the practice environment, there is a need for a skill mix with access to dietitians and mental health staff as well as support for staff in manageing and discussing difficult ethical dilemmas. When it comes to the person-centred processes, they need to be more focused on the older person and based on knowledge of the older person’s life history [14]. Given the shortcomings at the macro and prerequisite levels, and in the practice environment, it is understandable that staff express frustration. These shortcomings can also mean that the signs of EDs that do exist are not taken into account.

Participants in the present study expressed frustration that once someone is admitted to residential care, all their individual care needs are replaced by the one overreaching label of “old”. However, they did the same thing themselves when initially claiming that all of the residents’ problematic eating behaviors were caused by dementia or the ageing process. A 2022 scoping review on the causes of moral distress among care professionals working with older people showed that organizational restraints, such as lack of resources and inadequate care pathways, caused significant moral distress [41]. Since the participants of the current study expressed that they did not have adequate resources and did not know how to ensure that residents received specialized care, they may have coped by turning a blind eye to the problem by attributing the residents’ problematic eating behaviors to their age, thereby negating the need to facilitate specialized care access.

Earlier research has also found barriers to adequate nutritional care in the social, structural and ideological contexts both in residential care [42] and in hospital and home care [43]. With regard to the ethical dilemmas expressed in our study, previous research shows that ethical dilemmas frequently occur in nursing home settings, related to, for example, residents’ autonomy [44]. When it comes to ethical dilemmas related to nutrition and eating, most studies are related to end-of-life care [45, 46], or to artificial nutrition and hydration (enteral/parenteral nutrition) [47].

Our study breaks new ground in that it touches on ethical dilemmas specifically related to eating disorders and disordered eating behaviors, and to nutrition-related considerations in residents that may be cognitively impaired, but not seriously ill or at end-of-life. There is, as far as we know, no previous studies on eating disorders or disordered eating behaviors in residential care settings, and the present study offers novel insights into how staff misattribute symptoms of eating disorders as dementia or simply as a result of normal ageing. It also sheds light on staff’s sense of patients being excluded from specialised care pathways.

Future research should prioritise training and education interventions in order to upskill nursing home staff and provide them with the necessary tools to identify EDs and disordered eating behaviors among the residents. Whilst we have been unable to identify previous research on specific eating disorder education for nursing home staff, a recent scoping review found that structured, adaptable education interventions aimed at nursing staff in residential care resulted in improved nutrition knowledge and better nutrition status among the residents [48].

As this is a vulnerable group, any future research will need to carefully consider informed consent in order to safeguard autonomy and integrity. Any assessment methods to identify possible EDs will need to be carefully adapted to the individual’s abilities and conditions, and carried out together with relatives and those formal carers who are well-known to the older person.

### Methodological considerations

Methodologically, focus-groups interviews are well-suited for exploring consensus, diversity, and interaction patterns. Ensuring skilled moderation and incorporating systematic observation [38] enhances both the credibility and depth of the collected data. To ensure credibility, first and last authors listened to all audiotapes and reviewed the interview transcripts. Subsequently, the first and last authors discussed the analysis process at the coding stage. When it came to the formulation of themes, discussions included the second author, and the draft manuscript was then circulated for discussion among all authors. Furthermore, there was a variation in different professions in the focus groups, which might capture different experiences. Quotes are presented to exemplify the themes and variations within the themes as well as interactions between the focus group participants. Consensus on interpreting the participants’ experiences and reflections was reached among all authors, strengthening dependability. To further enhance dependability, focus group interviews and analyses were performed during a relatively short time period. The study reporting was guided by the Consolidated Criteria for Reporting Qualitative Studies (COREQ) checklist [39].

The present study is not without its potential limitations. Firstly, it is possible that the presence of first-line managers may have inhibited the other participants from openly voicing their opinions on problems or negative experiences. However, all participants seemed comfortable speaking openly during the focus groups, and there were no differences between the groups in terms of negative experiences and reflections shared.

Secondly, the number of participants varied between the groups, from 4 to 11. Eleven participants are above the generally recommended number for focus groups [38], and some participants may have felt overwhelmed and reluctant to speak in front of such a large group. As moderator, the first author made a conscious effort to give each participant time and space to speak.

Thirdly, all focus groups were conducted at nursing homes situated in roughly the same geographical location in mid-Sweden, meaning that transferability to other geographical settings may be limited.

Ensuring the trustworthiness of qualitative research is critical. Throughout the process, Graneheim and Lundman [36] emphasizes the importance of maintaining rigor through systematic procedures and transparency. This included documentation of coding decisions, and constant reflection on the researcher’s biases and assumptions. The final phase involved synthesizing the theme into a coherent narrative that provides a detailed and nuanced understanding of the research topic. This narrative is grounded in the data, with ample evidence provided to support the findings. Graneheim and Lundman [35] content analysis is characterized by its flexibility, allowing researchers to adapt the process to fit the specific context and goals of their study, while maintaining a rigorous approach to data analysis.

## Conclusions

In conclusion, the results of the present study show that based on the reflections of staff, disordered eating behaviors do occur in residential care settings, and staff generally feel unable to identify and treat them. This lack of knowledge and education constitutes a threat to the quality of life of older people in residential care, and poses a challenge to the provision of person-centred care.

Research on eating disorders and disordered eating behaviors among older people in general, and nursing home residents in particular, is very sparse, and further studies are urgently needed. Treatment for low appetite due to dementia or as a pharmaceutical side effect is not the same as treatment of an eating disorder, and it is vitally important that staff are given the tools to differentiate between these so that they are able to provide person-centred care and improve residents’ quality of life.

## Data Availability

The summary data are in the main document. Research data (the data set with individual data) are not shared. Individual data are not available due to general data protection regulations (GDPR).

## Abbreviations

ED: Eating disorder
FG: Focus group
PCC: Person-centred care
RN: Registered nurse
